# In Vitro Acid Resistance of Feline-Derived *Candidozyma auris* Isolates in Simulated Gastric Fluid: A Pilot Study

**DOI:** 10.3390/jof12080577

**Published:** 2026-08-04

**Authors:** Andrea Grassi, Patrizia Danesi, Sara Rigamonti, Sofia Sgubin, Paola Prati, Emanuela Olivieri

**Affiliations:** 1 Istituto Zooprofilattico Sperimentale della Lombardia e dell’Emilia Romagna, 27100 Pavia, Italy; sara.rigamonti@izsler.it (S.R.); paola.prati@izsler.it (P.P.); emanuela.olivieri@izsler.it (E.O.); 2 Istituto Zooprofilattico Sperimentale delle Venezie, 35020 Legnaro, Italy; pdanesi@izsvenezie.it (P.D.); ssgubin@izsvenezie.it (S.S.)

**Keywords:** *Candida auris*, in vitro digestion, acid resistance, cats, pH

## Abstract

The survival of *Candidozyma* (*Candida*) *auris* under gastric conditions remains poorly understood, particularly in veterinary species. This pilot in vitro study evaluated the resistance of two cat-derived *C. auris* strains and *Candida parapsilosis* ATCC 22019 in simulated gastric fluid (SGF). Yeast suspensions were exposed to SGF adjusted to pH 1–5 and 7 for up to 4 h at 37 °C, and colony-forming units (CFUs) were enumerated at 1, 2, and 4 h. Results were expressed as mean log10 CFU/mL ± standard deviation. A clear pH-dependent effect was observed: At pH 7, all strains showed increased CFU counts over time, whereas acidic conditions induced a marked reduction in culturability. At pH 1, reductions of approximately 3–4 log10 units occurred within 1 h, while at pH 2–5 a more moderate decrease of about 1 log10 unit was observed, with most reduction occurring early and stabilizing over time. Two-way ANOVA confirmed significant effects of pH and exposure time, with a significant interaction for all strains (*p* < 0.0001). Overall, gastric-like acidity strongly reduced, but did not completely abolish, the culturability of the two feline *C. auris* isolates under the specific in vitro conditions tested. Further studies using larger isolate collections and more physiologically relevant gastrointestinal models are needed to determine whether these findings translate into in vivo survival, intestinal transit, or colonization.

## 1. Introduction

Companion animals share close domestic and social environments with humans and may act as asymptomatic reservoirs of microorganisms with zoonotic potential. The detection of *Candidozyma* (*Candida*) *auris* in animal hosts is reshaping the current understanding of the ecology of this emerging multidrug-resistant yeast, traditionally considered a pathogen largely confined to humans, especially in healthcare settings.

*C. auris* has been isolated from different animal species, including dogs [1,2], a captive dolphin [3], locusts [4], and a cobra [5], suggesting that its ecological niche may extend beyond hospital environments. These findings raise the possibility that animals could serve as transient or persistent reservoirs and contribute to environmental dissemination.

A recently published case describing prolonged fecal shedding of *C. auris* in a cat further supports the hypothesis that this yeast may survive gastrointestinal transit and potentially colonize the gastrointestinal tract, where it may persist without causing invasive disease [6]. However, persistence in feces should not be interpreted as evidence of gastrointestinal colonization, infectivity, or transmission, all of which require direct investigation in dedicated models.

Gastric acidity represents a major barrier for microorganisms in the digestive tract, and, in addition to its digestive role, it contributes to the disinfection of ingested food and contaminated materials. In healthy cats, fasting gastric pH ranges from 1 to 2.7, whereas post-prandial pH may rise transiently depending on meal composition and physiological variability [7,8]. Similar fluctuations have been described in dogs and humans [9,10,11]. Accordingly, evaluating fungal culturability across a range of pH values may provide preliminary information on how animal-derived isolates respond to gastric-like acid stress under controlled laboratory conditions.

While the ability of *C. auris* to colonize skin and rectal sites in humans has been extensively investigated, information regarding its survival under gastric conditions is still limited, and evidence in animal species is scarce.

This pilot in vitro study evaluated the CFU-based culturability of two feline-derived *C. auris* isolates after exposure to simulated gastric fluid (SGF) at different pH values for up to 4 h. Because *C. auris* exhibits substantial inter-clade and intra-clade variability in stress tolerance, virulence, and environmental persistence [12], results obtained from a small number of animal isolates should be interpreted cautiously and not generalized to the species as a whole.

## 2. Materials and Methods

### 2.1. Candidozyma Strain Preparation and Cultivation

Two animal-derived strains of *Candidozyma auris* and *Candida parapsilosis* ATCC 22019 (control strain) were used in this study. Among the isolates available at the time of the study, only two *C. auris* strains of feline origin were known and could be tested. One strain was recovered from a cat with persistent fecal shedding and has been previously reported (GenBank accession PX112672) [6].

The second strain (GenBank accession PX113352) was obtained within the framework of a research project on fungal isolates from animals, during post-mortem screening of a feral cat that died from causes unrelated to *C. auris* infection, specifically following infection with feline parvovirus (FPV).

For the selective isolation of the yeast, samples were processed as previously described [6]. Briefly, fecal samples were processed within 4 h of collection and plated onto Selective Auris Medium (SAM) [13], followed by incubation at 41.5 °C for 7 days. Fungal colonies grown on SAM were subcultured onto Sabouraud Dextrose Agar (SDA) and incubated at 37 °C for 48 h. Isolates were identified by MALDI-TOF mass spectrometry (Bruker Biotyper^®^, Bruker Daltonics GmbH & Co. KG, Bremen, Germany) according to the manufacturer’s instructions. Molecular confirmation was performed by PCR amplification and sequencing of the ITS1 and ITS2 regions of the rDNA, as previously described [14].

Fresh cultures were obtained by subculturing isolates on Sabouraud Dextrose Agar (SDA) at 37 °C for 48 h, followed by inoculation into Yeast Peptone Dextrose (YPD) broth and incubation at 37 °C for 18 h to prepare viable cells for the experimental assay. Cell suspensions were then serially diluted 10-fold in phosphate-buffered saline (PBS), and fungal counts were determined by plating appropriate dilutions on PDA and incubating at 37 °C for 48 h for CFU enumeration.

### 2.2. Acid Resistance Assay

SGF was prepared according to the method described by Thomas et al. (2004) [15], with minor modifications to the hydrochloric acid (HCl) concentration to obtain different final pH values.

In the original protocol, SGF consisted of 0.084 N HCl, 35 mM NaCl, and 4000 U of pepsin, adjusted to pH 1.2 or 2.0 and pre-heated to approximately 37 °C prior to the addition of the test material. In the present study, the HCl concentration was adjusted to achieve final pH values of 1, 2, 3, 4, and 5 while maintaining pepsin activity at 4000 U/mL, following the experimental approach described by Ikeda et al. (2024) [16] for other *Candida* species. An additional condition at pH 7 was included as a neutral pH control to assess the influence of acidic conditions on the tested strains.

A 5% (*v*/*v*) yeast inoculum was added to 2 mL of SGF and incubated at pH values ranging from 1 to 7 for 4 h at 37 °C in 24-well plates.

Fungal survival under acidic conditions was assessed by plating ten-fold serial dilutions of the culture medium at 1, 2, and 4 h of incubation onto PDA, using 100 µL of each dilution per plate, followed by incubation at 37 °C for 48 h to determine CFU-based culturability. Each fungal strain was tested in two independent experiments, each performed in triplicate.

### 2.3. Statistical Analysis

Fungal strains were tested in two independent experiments performed on different days (biological replicates), and conditions within each experiment were plated in triplicate (technical replicates). For statistical analysis, CFU counts were log10-transformed, and the mean value of the technical triplicates from each biological replicate was used as the observational unit. For each strain, the effects of pH and exposure time on culturability were evaluated by two-way ANOVA including the interaction term, followed by an appropriate post hoc test for pairwise comparisons between pH groups. Data are presented as mean ± standard deviation (SD). Statistical significance was set at *p* < 0.05.

## 3. Results

**Figure 1:**
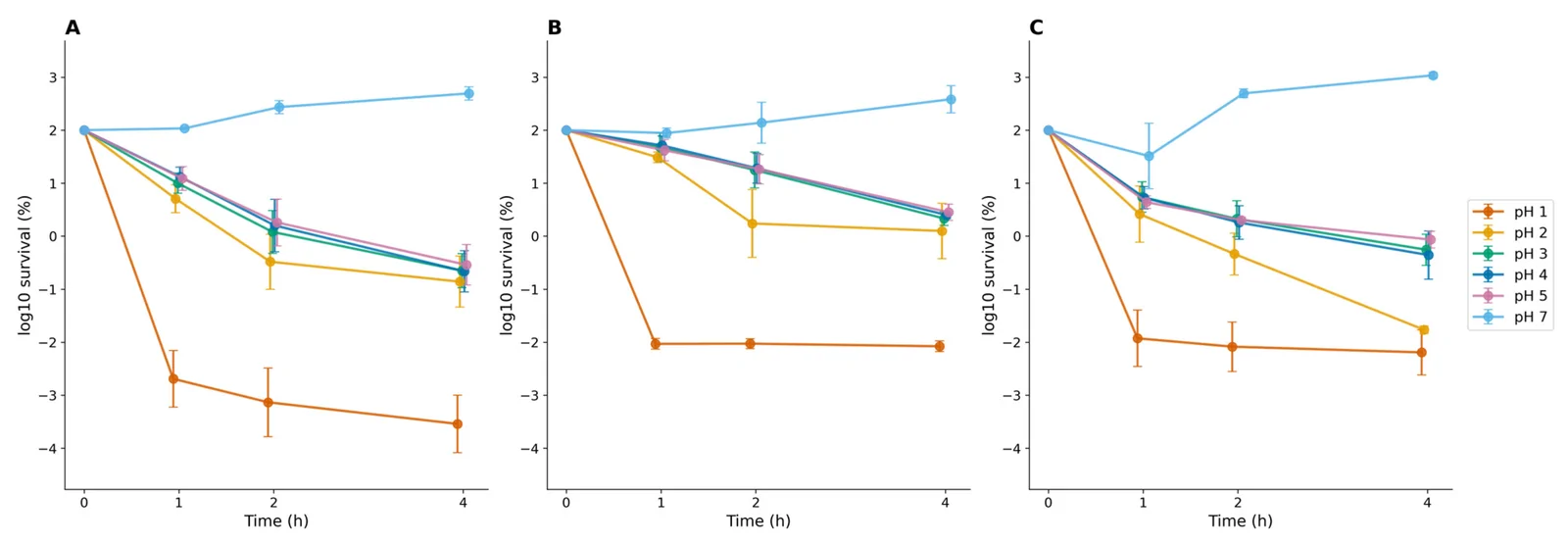
Time-dependent changes in culturable cell counts of yeasts exposed to simulated gastric fluid at different pH values. (**A**) feline *C. auris* isolate from a domestic cat; (**B**) feline *C. auris* isolate from a feral cat; (**C**) *C. parapsilosis* ATCC 22019. Survival is expressed as the percentage of the initial inoculum on a logarithmic scale. Data are presented as mean values with standard deviation (SD) of two biological replicates, each measured in technical triplicate. Two-way ANOVA showed significant effects of pH, exposure time, and their interaction for all three strains (*p* < 0.0001). Post hoc pairwise comparisons indicated that pH 1 resulted in significantly lower survival than pH 3–5 at all time points, whereas no significant differences were detected among pH 3, 4, and 5.

The survival kinetics of yeast strains were analyzed by calculating the mean log10 CFU/mL values from biological replicates. Raw CFU counts were converted to log10 scale to facilitate data normalization and comparison. For each pH condition and time point (0, 1, 2, and 4 h), the arithmetic mean and the standard deviation (SD) were calculated. Survival curves were plotted as mean log10 CFU/mL over time, with vertical error bars representing the standard deviation of the replicates, illustrating the variability across experimental runs (Figure 1). The corresponding numerical values for each strain, pH, and time point are reported in Tables S1–S3 (Supplementary Materials).

Exposure to SGF revealed a clear pH-dependent pattern for all strains. At pH 7, CFU counts increased over the 4 h incubation period for both feline *C. auris* isolates and for *C. parapsilosis*, whereas acidic conditions produced a time-dependent reduction in culturability. The greatest reductions were observed at pH 1, with an approximately 3–4 log10 decline occurring within the first hour. At pH 2–5, decreases were more moderate, generally around 1 log10, and tended to occur mainly during the early phase of incubation, followed by relative stabilization.

Two-way ANOVA demonstrated significant effects of pH and exposure time, as well as a significant interaction between these factors, for all three strains (*p* < 0.0001). Post hoc comparisons indicated that, for each strain and at all time points, pH 1 yielded significantly lower mean log10 CFU/mL values than pH 3–5, whereas no significant differences were detected among pH 3, 4, and 5 (Tables S1–S3, Supplementary Materials).

## 4. Discussion

This pilot study demonstrates that simulated gastric acidity markedly reduces, but does not abolish, the culturability of feline-derived *C. auris*. Even at pH 1, a measurable fraction of cells remained culturable after 4 h of exposure, indicating a degree of acid tolerance in a subset of the population, consistent with previous observations in other *Candida* species [16]. Under neutral conditions, all strains showed increasing CFU counts over time, confirming that growth capacity was retained in the absence of acid stress and that the reductions observed at low pH reflect a genuine stress response rather than baseline culture instability. The response observed in the two feline *C. auris* isolates may also reflect known stress-adaptation traits of this species. Previous studies have shown that *C. auris* can tolerate multiple physiologically relevant stresses and that its adaptive response involves regulators linked to cell wall remodeling, proton homeostasis, oxidative stress management, and heat-shock responses [17,18,19,20,21]. The present study did not investigate these mechanisms directly; therefore, any mechanistic interpretation remains hypothetical and should be addressed in future work using molecular or phenotypic assays.

Acid resistance is not unique to *C. auris*. Previous work has shown that *C. auris* grows optimally under mildly acidic to neutral conditions (pH 5–7), with growth kinetics comparable to *C. albicans*, but with reduced tolerance as pH decreases toward strongly acidic values [20]. This is consistent with the pattern observed here, where moderate reductions occurred at pH 3–5 while the most pronounced killing occurred only at pH 1. At intermediate pH values (pH 3–5), reductions in culturability were more limited and tended to stabilize over time. These conditions may partially approximate post-prandial states, during which gastric pH in cats can transiently increase. In contrast to the pathogenic *Candida* species examined by Ikeda et al., which showed slight increases in fungal burden under acidic SGF, the feline *C. auris* isolates tested here did not proliferate under any acidic condition, suggesting either a distinct stress-response profile or differences in strain background, inoculum preparation, and assay conditions between the two studies.

The partial survival observed here is consistent with the broader literature describing *C. auris* as an unusually stress-tolerant and environmentally persistent organism. Comprehensive reviews of *C. auris* biology have highlighted its capacity for prolonged survival on abiotic surfaces and its resilience across diverse ecological niches, a trait proposed to underlie both its nosocomial persistence and its emergence in non-clinical environments [19,20]. Experimental murine models have further shown that *C. auris* strains differ substantially in their gastrointestinal colonization capacity and fecal shedding intensity, with invasive strains producing significantly higher stool fungal burdens than non-invasive strains [22]. This strain-dependent variability aligns with the inter-clade and intra-clade heterogeneity previously reported for *C. auris* stress tolerance and virulence, suggesting that the two feline isolates tested here may not fully represent the range of acid-response phenotypes present within the species. Host-side factors regulating gastrointestinal colonization, including mucosal immune responses, have also been shown to modulate *C. auris* persistence in vivo, underscoring that our static SGF model captures only one component of a multifactorial process [23].

Murine intragastric challenge studies have demonstrated that *C. auris* can be recovered from feces, blood, liver, and kidney following gastrointestinal inoculation, indicating that gastric transit and subsequent dissemination are biologically plausible even without extensive local proliferation [22]. This mechanistic hypothesis offers a credible explanation for the persistent fecal shedding previously documented in a naturally infected cat, in which *C. auris* was recovered repeatedly despite the absence of clinical gastrointestinal disease [6]. Taken together, the in vitro acid tolerance observed here and the in vivo gastrointestinal recovery documented in mice and in the feline shedding case are compatible with, though not proof of, gastrointestinal passage as one possible route sustaining shedding.

These findings should be interpreted within the constraints of a pilot in vitro model. The assay does not reproduce intestinal transit, bile exposure, mucosal interactions, or microbiota competition, and therefore cannot establish colonization, infectivity, or transmission potential [24,25]. Conclusions are limited to survival of culturable cells under the specific SGF conditions tested. The isolate collection was restricted to two feline-derived strains, both recovered from fecal material, which precludes generalization across the substantial inter-clade and intra-clade variability reported for *C. auris*. Pepsin activity, although held at a constant concentration, varies with pH, so results reflect survival under SGF-like conditions differing in acidity rather than under equivalent enzymatic activity. Finally, plating without prior neutralization may have allowed residual acidity to affect colony recovery at low pH, potentially underestimating true survival. Given the proposed role of migratory birds in *C. auris* dissemination [26], future work should also examine yeast responses to simulated avian digestive temperatures (39–41 °C) [27].

Overall, these pilot findings position gastric acidity as a partial but incomplete barrier to *C. auris* viability, offering a mechanistic starting point for understanding how this stress-adapted yeast may persist within the feline gastrointestinal tract.

## 5. Conclusions

This pilot study shows that gastric-like acidity is a substantial but incomplete barrier to the culturability of feline-derived *C. auris*, with a measurable fraction of cells surviving even the most acidic condition tested. This partial acid tolerance offers a plausible mechanistic basis for the fecal persistence previously observed in a naturally shedding cat, and provides a starting point for future research rather than a definitive answer on gastrointestinal survival. Future studies should include a larger and genetically diverse isolate collection, direct comparison across clades, and more physiologically relevant gastrointestinal models to determine whether the acid tolerance observed here translates into genuine gastrointestinal colonization, shedding, or transmission risk in vivo.

The *Candidozyma auris* strains used for the study were deposited at the Biobank of Veterinary Resources-IZSLER (http://www.ibvr.org/ accessed on 31 July 2026) under the accession codes FUN LO SPV 11 (isolated from a domestic cat) and FUN LO SPV 14 (isolated from a feral cat).

## Data Availability

The original contributions presented in this study are included in the article; further inquiries can be directed to the corresponding author.

## Supplementary Materials

The following supporting information can be downloaded at https://www.mdpi.com/article/10.3390/jof12080577/s1, Figure S1: Representative agar plates of the three yeast strains after 1 h of incubation in simulated gastric fluid at pH 7. The top row shows the feline *Candidozyma auris* isolate from a feral cat, the middle row shows *Candida parapsilosis* ATCC 22019, and the bottom row shows the feline *C. auris* isolate from a domestic cat. Plates correspond to 10^−3^, 10^−2^, 10^−1^ dilutions and undiluted (“as is”) samples from left to right. Table S1: Mean log10 CFU/mL ± SD for the domestic cat-derived *Candidozyma auris* isolate at different pH values and incubation times. Table S2: Mean log10 CFU/mL ± SD for the feral cat-derived *Candidozyma auris* isolate at different pH values and incubation times. Table S3: Mean log10 CFU/mL ± SD for *Candida parapsilosis* ATCC 22019 at different pH values and incubation times. Table S4: Two-way ANOVA (pH × time) on log10-transformed survival percentages indicated highly significant effects of pH, time and their interaction for all three isolates (all *p* < 0.0001).

## Abbreviations

The following abbreviations are used in this manuscript:

ATCC	American Type Culture Collection
YPD	Yeast Peptone Dextrose
PDA	Potato Dextrose Agar
PBS	Phosphate-Buffered Saline
SGF	Simulated Gastric Fluid
CFU	Colony-Forming Units
SDA	Sabouraud Dextrose Agar
