# Supplementary material for: In Vitro Acid Resistance of Feline-Derived Candidozyma auris Isolates in Simulated Gastric Fluid: A Pilot Study
Source: J Fungi (Basel). 2026 Aug 4;12(8):577. doi: 10.3390/jof12080577 (PMC13514687; doi:10.3390/jof12080577)
Supplement: Supplementary file 1 [file jof-12-00577-s001.zip › jof-4448859-supplementary.pdf]

Supplementary Materials

Data are expressed as mean log10 CFU/mL ± standard deviation (SD) of two independent experiments (biological replicates) for each pH and time point, each experiment performed in technical triplicate.

pH	Time (h)	Mean log10 CFU/mL	SD
1	0	6.12	0.10
1	1	2.10	0.01
1	2	2.10	0.15
1	4	2.05	0.13
2	0	6.12	0.10
2	1	5.61	0.03
2	2	4.36	0.73
2	4	4.22	0.57
3	0	6.12	0.10
3	1	5.80	0.25
3	2	5.37	0.33
3	4	4.46	0.04
4	0	6.12	0.10
4	1	5.83	0.13
4	2	5.41	0.26
4	4	4.52	0.05
5	0	6.12	0.10
5	1	5.75	0.15
5	2	5.39	0.25
5	4	4.58	0.08
7	0	6.24	0.27
7	1	6.19	0.16
7	2	6.53	0.21
7	4	6.83	0.06

Table S1. Mean log10 CFU/mL ± SD for the domestic cat-derived *Candidozyma auris* isolate at different pH values and incubation times.

pH	Time (h)	Mean log10 CFU/mL	SD
1	0	6.12	0.10
1	1	2.10	0.01
1	2	2.10	0.15
1	4	2.05	0.13
2	0	6.12	0.10
2	1	5.61	0.03
2	2	4.36	0.73
2	4	4.22	0.57
3	0	6.12	0.10
3	1	5.80	0.25
3	2	5.37	0.33
3	4	4.46	0.04
4	0	6.12	0.10
4	1	5.83	0.13
4	2	5.41	0.26
4	4	4.52	0.05
5	0	6.12	0.10
5	1	5.75	0.15
5	2	5.39	0.25
5	4	4.58	0.08
7	0	6.24	0.27
7	1	6.19	0.16
7	2	6.53	0.21
7	4	6.83	0.06

Table S2. Mean log10 CFU/mL ± SD for the feral cat-derived *Candidozyma auris* isolate at different pH values and incubation times.

pH	Time (h)	Mean log10 CFU/mL	SD
1	0	5.95	0.38
1	1	2.03	0.29
1	2	1.87	0.20
1	4	1.77	0.14
2	0	5.95	0.38
2	1	4.37	0.30
2	2	3.62	0.11
2	4	2.19	0.36
3	0	5.95	0.38
3	1	4.71	0.05
3	2	4.29	0.04
3	4	3.70	0.01
4	0	5.95	0.38
4	1	4.68	0.13
4	2	4.22	0.01
4	4	3.60	0.20
5	0	5.95	0.38
5	1	4.59	0.25
5	2	4.25	0.34
5	4	3.89	0.19
7	0	5.64	0.05
7	1	5.16	0.73
7	2	6.34	0.01
7	4	6.68	0.08

Table S3. Mean log10 CFU/mL ± SD for *Candida parapsilosis* ATCC 22019 at different pH values and incubation times.

Isolate	Factor	F-value	p-value
C. parapsilosis ATCC 22019	pH	312.67	$p < 0.0001$
C. parapsilosis ATCC 22019	time	30.50	$p < 0.0001$
C. parapsilosis ATCC 22019	pH × time	19.07	$p < 0.0001$
C. auris FERAL CAT	pH	479.05	$p < 0.0001$
C. auris FERAL CAT	time	68.59	$p < 0.0001$
C. auris FERAL CAT	pH × time	16.65	$p < 0.0001$
C. auris DOMESTIC CAT	pH	371.70	$p < 0.0001$
C. auris DOMESTIC CAT	time	77.45	$p < 0.0001$
C. auris DOMESTIC CAT	pH × time	9.15	$p < 0.0001$

Table S4.Two-way ANOVA (pH × time) on log10-transformed survival percentages indicated highly significant effects of pH, time and their interaction for all three isolates (all $p < 0.0001$).

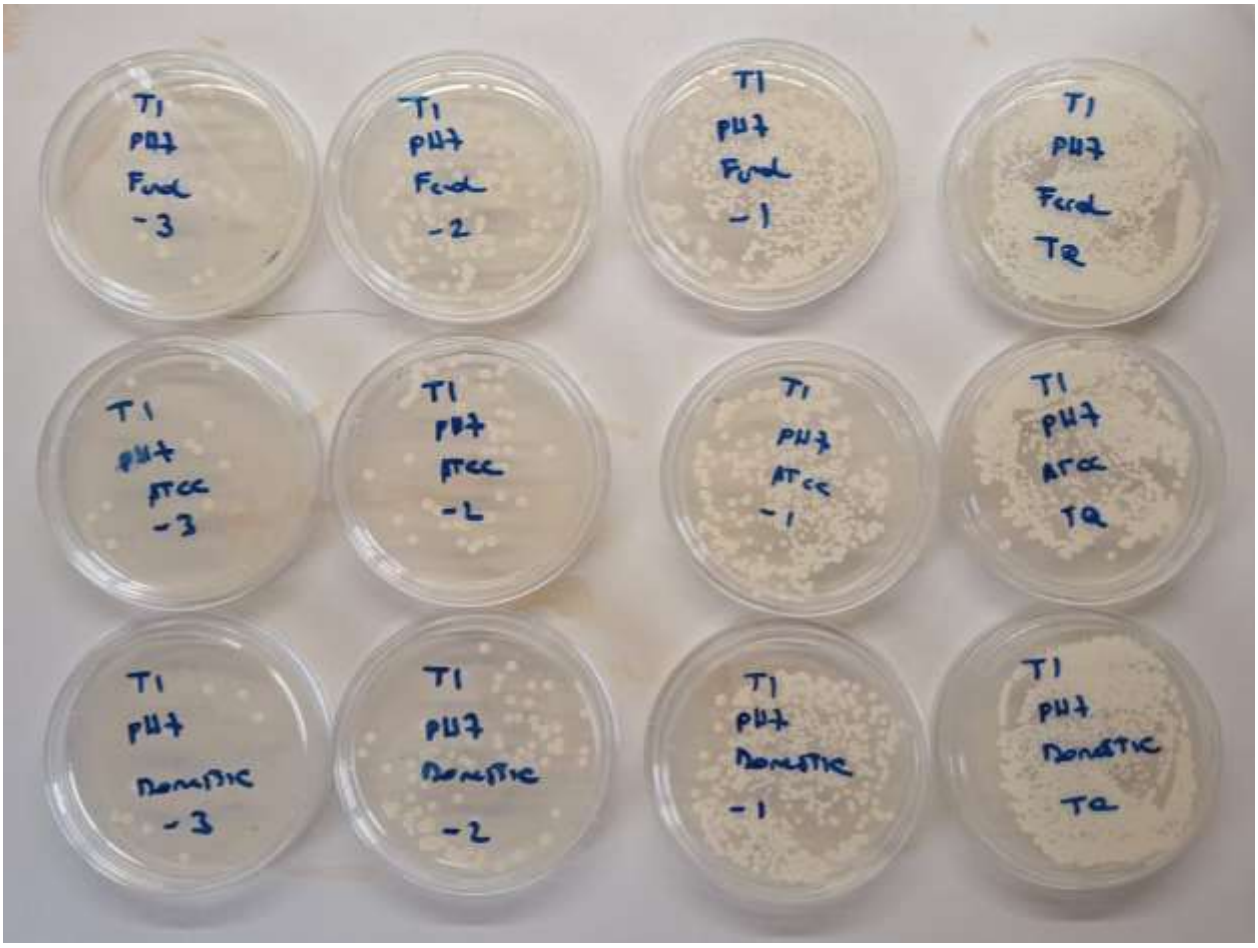

Figure S1. Representative agar plates of the three yeast strains after 1 h of incubation in simulated gastric fluid at pH 7. The top row shows the feline *Candidozyma auris* isolate from a feral cat, the middle row shows *Candida parapsilosis* ATCC 22019, and the bottom row shows the feline *C. auris* isolate from a domestic cat. Plates correspond to 10^{-3}, 10^{-2}, 10^{-1} dilutions and undiluted ("as is") samples from left to right.
